# Reducing *Igf-1r* Levels Leads To Paradoxical and Sexually Dimorphic Effects in HD Mice

**DOI:** 10.1371/journal.pone.0105595

**Published:** 2014-08-20

**Authors:** Silvia Corrochano, Maurizio Renna, Georgina Osborne, Sarah Carter, Michelle Stewart, Joel May, Gillian P. Bates, Steve D. M. Brown, David C. Rubinsztein, Abraham Acevedo-Arozena

**Affiliations:** 1 MRC Mammalian Genetics Unit, Harwell, Oxfordshire, United Kingdom; 2 Department of Medical Genetics, Cambridge Institute for Medical Research, University of Cambridge, Wellcome/MRC Building, Addenbrooke’s Hospital, Cambridge, United Kingdom; 3 Department of Medical and Molecular Genetics, King’s College London, London, United Kingdom; Institut Curie, France

## Abstract

Many of the neurodegenerative diseases that afflict people in later life are associated with the formation of protein aggregates. These so-called “proteinopathies” include Alzheimer’s disease (AD) and Huntington’s disease (HD). The insulin/insulin-like growth factor signalling (IIS) pathway has been proposed to modulate such diseases in model organisms, as well as the general ageing process. In this pathway, insulin-like growth factor binds to insulin-like growth factor receptors, such as the insulin-like growth factor 1 receptor (IGF-1R). Heterozygous deletion of *Igf-1r* has been shown to lead to increased lifespan in mice. Reducing the activity of this pathway had benefits in a HD *C. elegans* model, and some of these may be attributed to the expected inhibition of mTOR activity resulting in an increase in autophagy, which would enhance mutant huntingtin clearance. Thus, we tested if heterozygous deletion of *Igf-1r* would lead to benefits in HD related phenotypes in the mouse. Surprisingly, reducing *Igf-1r* levels led to some beneficial effects in HD females, but also led to some detrimental effects in HD males. Interestingly, *Igf-1r* deficiency had no discernible effects on downstream mTOR signalling in HD mice. These results do not support a broad beneficial effect of diminishing the IIS pathway in HD pathology in a mammalian system.

## Introduction

Huntington’s disease (HD) is an autosomal dominant, progressive, fatal, neurodegenerative disorder caused by an expanded CAG tract (polyglutamine or PolyQ) in exon one of the huntingtin gene [1]. The length of the polyQ tract is directly associated with the age-of onset signs of disease [2]. The major symptoms that characterize the disease are motor (chorea) and cognitive (behavioural mood changes, memory lapses and depression). There are no current treatment to cure Huntington’s disease [3].

The huntingtin protein is ubiquitously expressed, and appears to function in a variety of cellular processes from transport to apoptosis [4]. The mutation confers a toxic gain of function on the protein, which becomes aggregate-prone, leading to intracellular aggregates that are the hallmark of the disease [5]. A possible loss of function component is also associated with HD pathology [6]. Many of the neurodegenerative diseases that afflict people in later life are associated with the formation of intraneuronal or extraneuronal protein aggregates. These proteinopathies include Alzheimer’s disease (AD), Parkinson’s disease and conditions caused by polyglutamine tract expansion mutations, like HD. While the pathological hallmark of these proteinopathies is the presence of large protein aggregates, the most toxic species may be oligomers, with the aggregation process itself being an important factor [7]. A range of possible strategies for tackling proteinopathies have been proposed. On the one hand, one can try to decrease the levels of the toxic protein. This could be achieved by enhancing the degradation of cytoplasmic aggregate-prone proteins by for example inducing autophagy [8]–[10]. Indeed, autophagy-inducing drugs and genes can alleviate the toxicity of mutant huntingtin in a range of models [9], [11]. Another strategy may be to decrease the rate of protein aggregation. This may be possible by up-regulating chaperones like HSP70 via the HSF-1 transcription factor, to enhance productive and non-toxic protein folding [12], although the ability of HSF1 to up regulate this pathway has been shown to decrease with disease progression in HD mice [13].

One pathway that has attracted considerable attention for its ability to modulate proteotoxicity is the insulin/insulin-like growth factor signalling pathway (IIS). In this pathway, insulin-like growth factor (IGF-1) binds to insulin-like growth factor receptors, such as the insulin-like growth factor 1 receptor (IGF-1R), resulting in their activation. The tyrosine kinase activities of these receptors phosphorylate signalling molecules, including important effectors such as the insulin receptor substrate (IRS) protein family. Once phosphorylated, the IRS proteins act as molecular adaptors to facilitate downstream signalling pathways via protein kinase B or AKT (PKB/AKT), which serves as a major downstream effector of IIS signalling.

IIS has been shown to be altered in several neurodegenerative disorders [14]–[16]. The phosphorylation of AKT is found to be upregulated in many neurodegenerative diseases which could indicate a compensatory response to maximize IGF-1 signalling in these disorders [17]. On the other hand, a prolonged activation of IIS may also lead to a maladaptive response in HD [18]. AKT levels have also been found to be reduced in a rat model of HD and HD patients [19]. Activated AKT can have beneficial effects in neurodegeneration by activating anti-apoptotic pathways. Indeed, stimulation of IGF-1/AKT has been shown to be neuroprotective in HD through direct phosphorylation of huntingtin [17], [20] and arfaptin 2 [21], leading to amelioration of HD toxicity in cellular [22] and animal models [23]. Moreover, IGF-1 treatment (stimulating IIS) prevented age-related body weight loss in a HD mouse model, with no differences in motor behaviour, but restoring blood insulin levels [24].

In the opposite direction, IIS inhibition has been shown to ameliorate the proteotoxicity of different aggregate-prone proteins in *C elegans* models, including mutant huntingtin and beta-Amyloid (Abeta) [25], [26]. This protection was recently confirmed in AD mouse models [27], [28]. Cohen et al found that the heterozygous deletion of *Igf-1r* reduced Abeta induced behavioural impairment in mice, while correlating with the formation of denser soluble amyloid oligomers. One potential mechanism to explain this protection may be by the induction of autophagy due to diminished mTOR activation via AKT. Indeed, numerous studies have suggested that IGF-1 blocks autophagy via mTOR complex 1 (mTORC1) [29]–[31]. However, in a recent study we have shown that in the long term IGF-1R inhibition leads *in vitro* and *in vivo* to diminished autophagy by reducing the rate of autophagosome precursor formation at the plasma membrane in an mTORC2- and endocytosis-dependent manner [32].

Hence, based on previous data from different model organisms, reducing IIS may lead to both beneficial and deleterious consequences in HD. We thus tested the consequences of the heterozygous deletion of *Igf-1r* in a HD mouse model. We felt this was an important scenario to investigate, since the excess amyloid beta in AD is predominantly extracellular, while in HD, mutant huntingtin is intracellular. We considered that decreased *Igf-1r* levels in heterozygous null mice would be more relevant to potential therapeutic scenarios, given the lethality of a complete *Igf-1r* null background. Interestingly, the behavioural and pathological consequences of reducing IIS in HD mice were equivocal.

## Results

### Heterozygous deletion of IGF-1R has paradoxical effects in HD mice

Our initial experiments examined whether reduction of *Igf-1r* affected the classical phenotypes of an HD mouse model expressing the mutant fragment of the human huntingtin gene with 82 expanded polygutamine repeats (hemizygous N171-82Q). We examined heterozygous Knock Out (KO) *Igf-1r* mice, since adult mice with complete deletion of this gene are not viable [33]. To produce all relevant genotypes in one generation, we crossed congenic C57BL6/J N171-82Q HD mice with C57BL6/J *Igf-1r^+/−^* mice. In order to examine not only the age at the onset of the symptoms, but also the disease progression, we carried out a battery of behavioural test every two weeks, (modified SHIRPA analysis, see methods), together with motor testing via rotarod and grip-strength and weight measurements. We started at 8 weeks of age, before the onset of the symptoms, and stopped the phenotyping at 22 weeks, after which we left mice to reach their humane end-points to analyse survival. As different HD endophenotypes are differentially affected by sex in mice [34], [35], we separated data from males and females.

**Figure 1:**
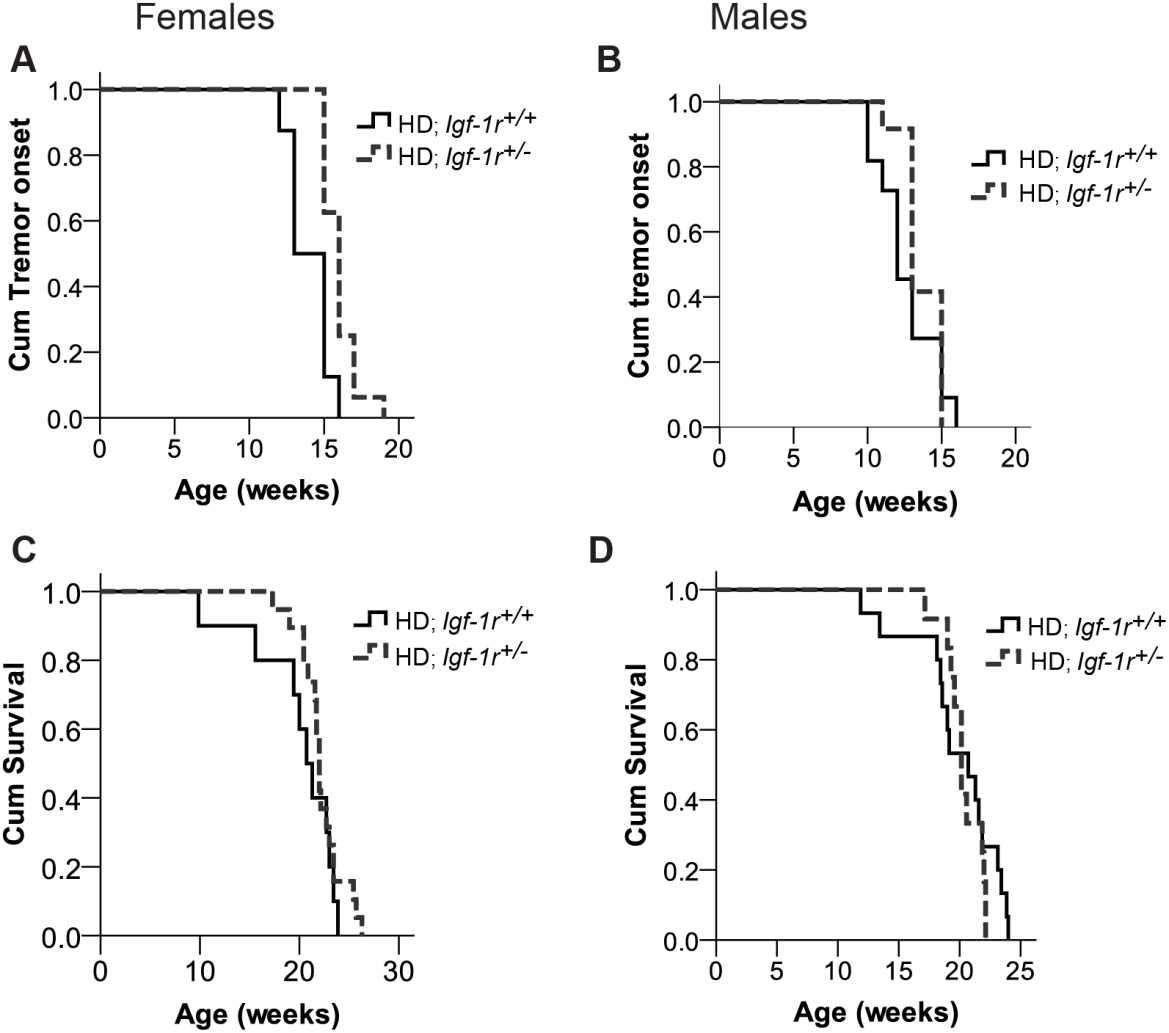
*Igf-1r* deficiency delays tremor onset in HD females without significantly affecting HD males or overall survival. **A–B.** Tremor onset was estimated via SHIRPA analysis on at least 8 mice per genotype per sex and time point. Tremor onset is significantly reduced in females (**A**) (average onset HD; *Igf-1r^+/−^*: 16.3 weeks compared HD; *Igf-1r^+/+^*: 14.1 weeks, p(Log-rank) = 0.002), but not in males (**B**) HD; *Igf-1r^+/−^*: 13.6 weeks compared HD; *Igf-1r^+/^* ^+^: 12.6 weeks, p(Log-rank) = 0.278). **C–D**. Time to reach end-stage (survival) of the N171-82Q HD mice is not modified by *Igf-1r* status. Survival was measured on at least 10 mice per sex and genotype. No significant differences were observed when comparing survival between HD; *Igf-1r^+/^* ^+^ and HD; *Igf-1r^+/−^* in female (**C,** n = 10 and 19 respectively, p (Log-rank) = 0.26) or males (**D,** n = 15 and 12 respectively**,** p(Log-rank) = 0.48), or when both sexes were combined p(Log-rank) = 0.79.

The development of tremors is a progressive endophenotype that appears in all HD mouse models. We measured tremor onset as part of the SHIRPA analysis in HD mice (non-HD mice have no tremors). In females, tremor onset was delayed in heterozygous *Igf-1r* HD mice (HD; *Igf-1r^+/−^*) when compared to controls (HD; *Igf-1r* ^+/+^ mice) (Figure 1A; p = 0.002). The average tremor age at onset in females was 16.3 weeks for HD; *Igf-1r* ^+/*−*^ versus 14.1 weeks for controls. However, tremor onset was not significantly different between control and *Igf-1r* deficient HD males (Figure 1B; p = 0.278). Thus, *Igf-1r* deficiency has no significant effect in HD males, but significantly delays the onset of this critical HD endophenotype in HD females. Previous data shows that heterozygous *Igf-1r* null female mice (*Igf-1r^+/−^)* live longer than littermate controls, while no significant lifespan differences were seen in males [33], [36]. When fed *ad libitum* on a standard diet and maintained in regular housing until reaching their humane end-points, *Igf-1r* deficiency did not significantly affect survival defined as time to reach the humane end-points for either male or female HD mice (Figure 1C, D).

**Figure 2:**
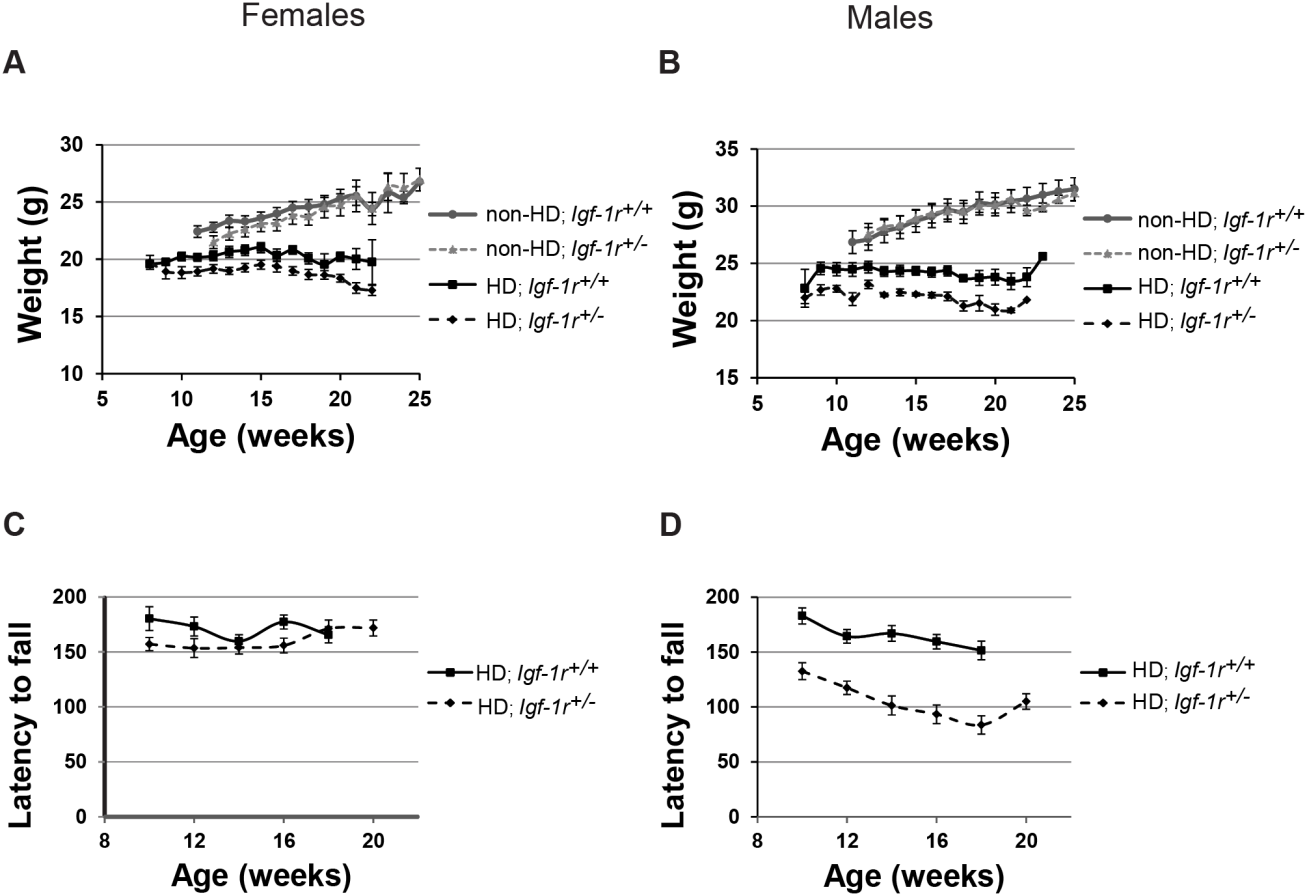
*Igf-1r* hemizygosity differentially affects body weight and rotarod performance in HD males and females. **A, B.** Body weights of at least 8 mice per genotype per sex and time point were scored. There were no significant weight differences between HD; *Igf-1r^+/+^* and HD; *Igf-1r^+/−^* female mice (**A**), except the 13 week time point. Male HD; *Igf-1r^+/−^* mice showed a significantly reduced body weight from the 9 week time point onwards (**B**). No differences between Non-HD; *Igf-1r^+/+^* and Non-HD; *Igf-1r^+/−^* mice. **C, D**. A reduced Rotarod performance was observed in HD; *Igf-1r^+/−^* compared to HD; *Igf-1r^+/+^* males (**C**), whereas no significant differences were observed in the female groups (**D**). No differences were observed between Non-HD; *Igf-1r^+/+^* and *Igf-1r^+/−^* mice (data not shown). Error bars represent 1×s.e.m (standard error of the mean).

In our C57BL/6J background, we found no significant differences in body weight between Non-HD; *Igf-1r^+/+^* and Non-HD; *Igf-1r^+/−^* males or females from 12 weeks of age (Figure 2A, B). However, in HD male mice, *Igf-1r* deficiency resulted in significantly reduced body weight in HD; *Igf-1r^+/−^* when compared to HD controls (Figure 2B; from 9 weeks of age onwards, p<0.05). Interestingly, *Igf-1r* deficiency did not significantly affect the weights of HD female mice at any stage (Figure 2A). Thus, *Igf-1r* deficiency has only a significant deleterious effect on the weight of HD males.

In agreement with the body weight phenotypes, *Igf-1r* deficiency significantly impaired rotarod performance on HD male mice, but had no significant effects on HD females (Figures 2C, 2D); no significant effects were present when comparing *Igf-1r* deficient Non-HD male or female mice (data not shown). When both sexes are combined, the overall differences between HD genotypes are significant for both weight and rotarod performance (Table S1). Grip strength was also assessed, but no significant differences were found in neither male nor female HD mice (Figure S1A, S1B).

Overall, *Igf-1r* deficiency had no significant effects on lifespan or grip-strength in HD mice, but on affected endophenotypes had opposite effects in male and female mice: In HD females, *Igf-1r* deficiency was neutral (rotarod, weight) or beneficial (delayed tremor onset), whereas in HD males had neutral (tremor onset) or detrimental effects (worsened rotarod performance and weight loss).

### Circulating levels of IGF-1 are differentially regulated in HD males and females at 12 weeks of age

**Figure 3:**
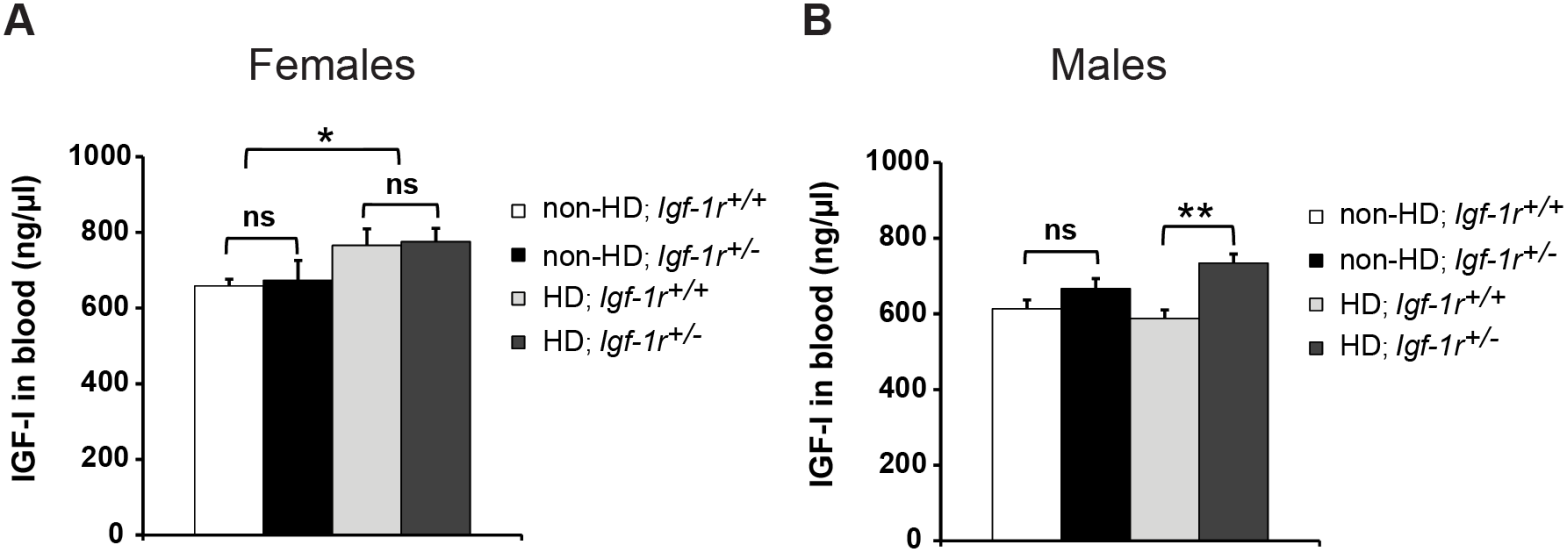
Serum levels of Igf-1 and glucose differ between male and female HD mice. **A, B.** Blood serum from fasted mice at 12 weeks of age (more than 4 mice per group). IGF-1 circulating levels measured by ELISA in females (**A**) and males (**B**) comparing the four possible genotypes. Error bars represent 1×s.e.m. *P<0.05; **P<0.01. ns = non-significant.

A possible explanation for the paradoxical effect observed above could be that males and females present different circulating levels of IGF-1. As a consequence of having less IGF-1 receptor, mice tend to compensate by increasing the levels of circulating IGF-1 [33]. We measured IGF-1 levels in blood and found no significant differences between Non-HD mice at 12 weeks of age (Figure 3A, 3B). For HD mice, we again found sexual dimorphism in the levels of circulating IGF-1. In females, IGF-1 levels were higher in HD mice compared to Non-HD controls (Figure 3A, p = 0.02), but *Igf-1r* deficiency had no effect and thus HD; *Igf-1r^+/+^* and HD; *Igf-1r^+/−^* females had similar amounts of circulating IGF-1. However, in males, the HD transgene alone could not significantly elevate IGF-1 levels; upregulation of circulating IGF-1 levels was found only in *Igf-1r* deficient HD males (Figure 3B, p = 0.002 comparing HD; *Igf-1r^+/+^* to HD; *Igf-1r^+/−^*). Remarkably, glucose levels in serum have a tendency to correlate with the IGF-1 levels (Figure S2A, S2B). Thus, circulating IGF-1 levels are differentially regulated in HD males and females at 12 weeks of age, with *Igf-1r* deficiency only significantly affecting IGF-1 levels in HD males. These differences in circulating IGF-1 levels paralleled the sexual behavioural differences and may contribute towards the opposite effects of *Igf-1r* deficiency in HD males and females.

### IGF-1R deficiency does not affect mutant huntingtin protein levels or overall aggregate numbers but modulate aggregate size

*Igf-1r* deficiency has been shown to affect mutant huntingtin aggregation in multiple models. One of our initial hypotheses was that loss of one copy of *Igf-1r* gene would result in decreased mTORC1 activity, which would increase autophagy and enhance removal of mutant huntingtin, as previously showed when inhibiting mTOR with rapamycin treatment [11], [37].

**Figure 4:**
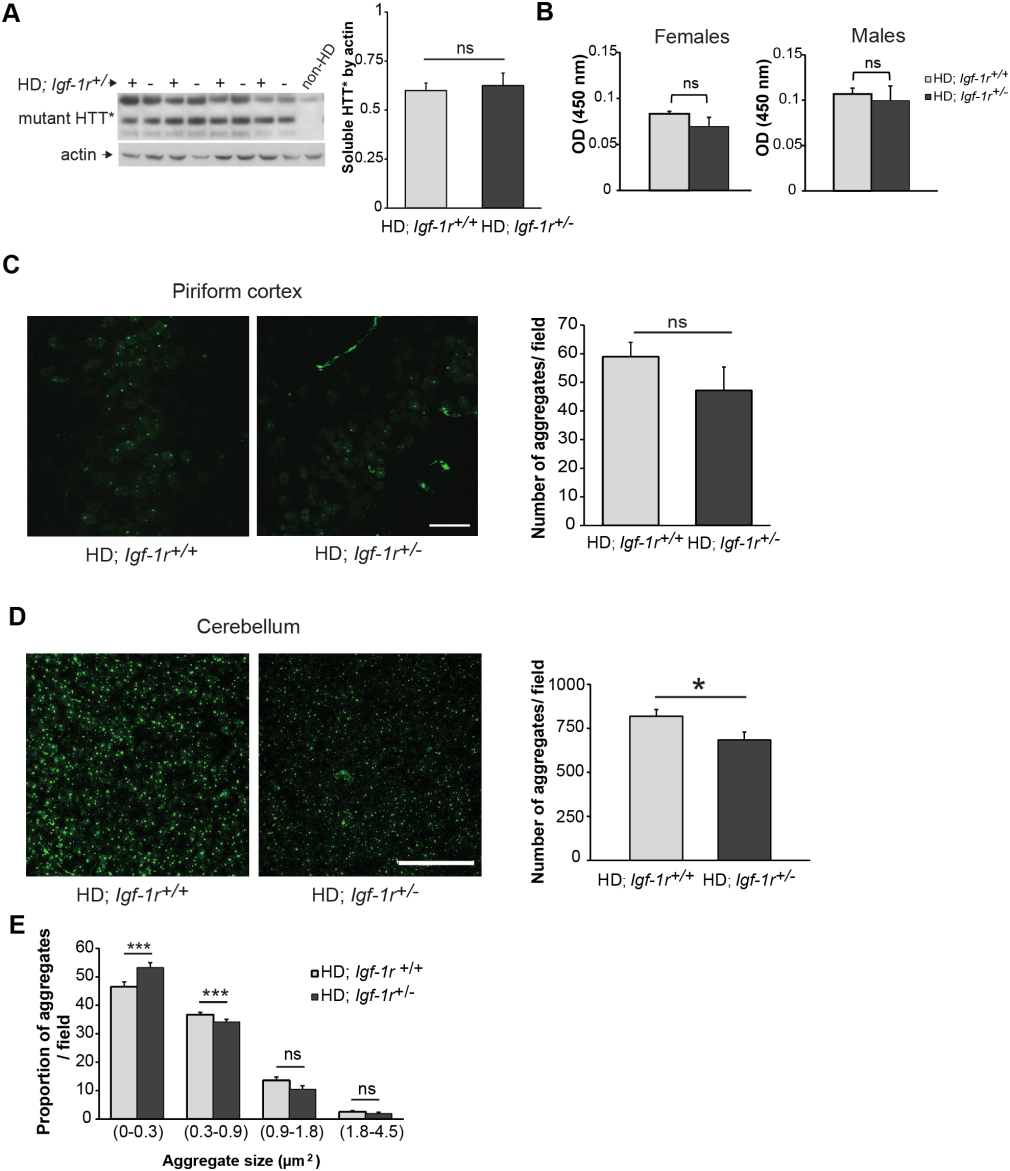
*Igf-1r* hemizygosity has minimal effect on the levels of mutant huntingtin protein. **A.** Female half-brain homogenates at 12 weeks of age of the indicated genotypes were subject to western blot analysis to measure soluble mutant huntingtin levels (HTT*). The corresponding graph represents the comparison of the densitometry analysis of HTT* levels relative to actin between HD; *Igf-1r^+/+^* and HD; *Igf-1r^+/−^* groups (n = 4 per genotype; p = 0.67). **B.** Using Microsens Aggregate Purification ELISA we measured the mutant HTT aggregates in homogenised cerebellums of 12 weeks old male and female HD mice (n = 4 per group and gender). No significant differences were found between female HD (p = 0.29) or male HD mice (p = 0.69). **C.** Quantification of huntingtin inclusions in the piriform cortex. Brain slices obtained from 13 weeks old female mice of the indicated genotypes were stained to detect the mutant huntingtin aggregates. Confocal images of the piriform cortex were obtained and analysed as detailed in the methods section. (n = 4 per HD group; p = 0.06). Scale bar represents 25 µm **D.** Quantification of huntingtin inclusions in the cerebellum of HD female mice. Representative images of confocal projections from the granular layer of cerebellum containing mutant huntingtin aggregates. The quantification of the total number of mutant huntingtin inclusions in the cerebellum shows a modest decreased in the number of inclusions found in the HD; *Igf-1r^+/−^* group compared to the HD; *Igf-1r^+/+^* (n = 6 per HD group; p = 0.019). Scale bar represents 50 µm. **E**. Automated quantification of images from D according to aggregate size reveal an increase in the percentage of small-size aggregates (0–0.3 µm^2^) coupled with a decreased in the percentage of medium-sized aggregates (0.3–0.9 µm^2^) in HD; *Igf-1r^+/−^* cerebellum compared to controls (p<0.001). Data are expressed as mean ± 1×s.e.m. *P<0.05; **P<0.01; ***P<0.001. ns = non-significant.

We thus evaluated both, the levels of mutant huntingtin protein in brain homogenates and the inclusion depositions in brain slices from HD females, as the slight beneficial phenotypical effects were only observed in mice of this gender. We first compared soluble huntingtin levels in the HD; *Igf-1r^+/−^* mice with that in controls by western blots from whole brain lysates at 12 weeks of age, but no differences were found (Figure 4A, p = 0.6). In the N171-82Q HD mouse model, inclusions are much more abundant in the cerebellum than in the cortex or striatum. We then measured mutant huntingtin in cerebellum using quantitative ELISA [38] in both male and female HD controls and *Igf-1r* deficient mice, and found no significant differences (Figure 4B, p = 0.29 for females and p = 0.69 for males).

We then quantified the number of inclusions formed in the piriform cortex and cerebellum of these mice. The chosen time point criteria was dictated by the age of onset of tremors in the HD; *Igf-1r* ^+/+^ group of females, which was around 13.5 weeks of age. In the piriform cortex at 13 weeks of age, compared to HD controls, the number of huntingtin inclusions had a trend towards being lower in *Igf-1r* deficient females (Figure 4C, p = 0.06). We thus counted overall inclusion numbers and also classified the inclusions according to size in cerebellum slices from HD females using a commercial automated analysing system (as detailed in the methods section). The total number of aggregates was significantly diminished in HD; *Igf-1r^+/−^* females when compared to littermate controls (Figure 4D, p = 0.019). Aggregates were then classified as small (in the range of 0 to 0.31 µm^2^), medium (0.32 to 0.9 µm^2^), large (0.91 to 1.8 µm^2^) and larger (1.81 to 4.5 µm^2^). Overall, small and medium inclusions are the major species in the cerebellum at this stage (13 weeks of age). Represented as percentage of total inclusions, big inclusions (large and larger) were similar between the genotypes. However, when compared to controls, the percentage of small aggregates in *Igf1-r* deficient HD females was significantly increased (Figure 4E, p = 0.001). This, taken together with a lower number of medium aggregates in HD; *Igf-1r^+/−^* than in controls (p<0.001) suggest that *Igf-1r* deficiency may have an effect on the formation and growth of cerebellar huntingtin aggregates.

In this context, it is possible to speculate that *Igf-1r* levels might influence huntingtin oligomerization and/or aggregation kinetics, as previously proposed for the Alzheimer’s disease-linked human peptide, Abeta [27]. For instance, this decrease in inclusion numbers, observed at 13 weeks of age, coincides with the delay in tremor onset seen in HD; *Igf-1r* ^+/*−*^ females, although it has no discernible effect on survival or other motor phenotypes assessed in HD mice.

### IGF-1R inhibition does not modulate mTOR signalling or autophagosome numbers in HD brains

Our data above suggest that *Igf-1r* deficiency is not sufficient to affect mutant huntingtin levels in brain or cerebellum, although it may have an effect on cerebellar inclusions in females. We thus decided to explore how *Igf-1r* deficiency affected downstream AKT and mTOR activation just before the age of phenotypic onset in female mice (12 weeks of age). *Igf-1r* would result in decreased mTOR activity, which would increase autophagy and enhance removal of mutant hungtingtin. The slight effect observed on the removal of mutant protein by *Igf-1r* deficiency in female brains could indicate that the expected mTORC1 activity, or downstream mTOR signalling, might not be as initially expected.

**Figure 5:**
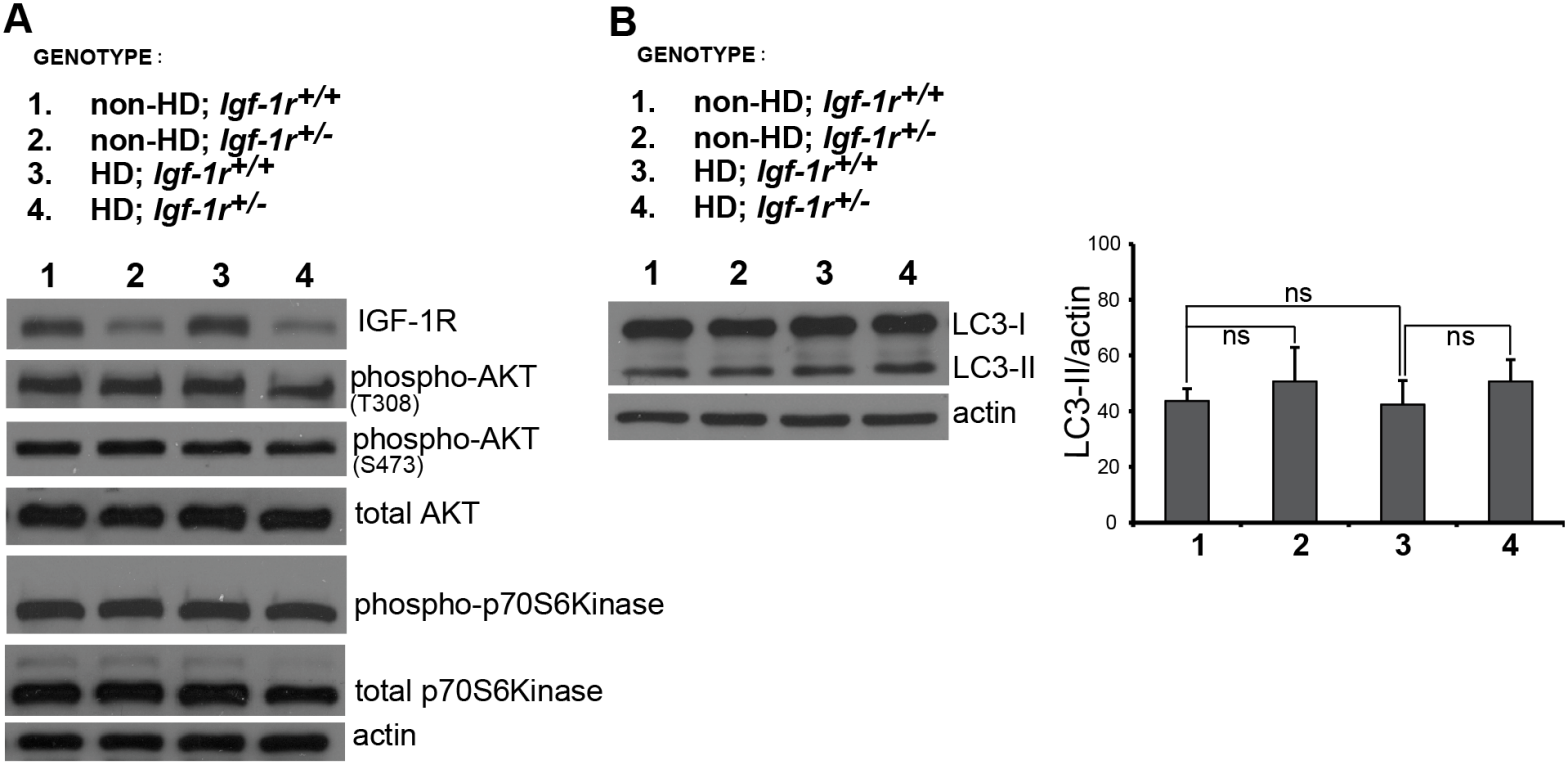
*Igf-1r* hemizygosity has no effect on AKT and S6K activation or autophagosome numbers in HD mice. **A.** *Igf-1r* deficiency leads to a significant decrease in IGF-1R protein levels (p = 0.0057) but does not significantly affect AKT phosphorylation at Ser473 or Thr308, nor the phosphorylation of S6K at Threonine 389 in half-brain homogenates of 12 weeks old female mice of the indicated genotypes. Representative images are presented. For densitometry analysis, four different samples for each genotype were used (n = 4 per group). Further analysis is presented in Figure S3. **B.** *Igf-1r* hemizygosity does not affect autophagosome numbers. Brain homogenates from 12 week-old females of the indicated genotypes were subjected to western blot analysis to assess LC3 levels. The graph reports the quantitative analysis of LC3-II levels relative to actin from four different brain samples for each genotype. The *p* values for the densitometric analyses were determined by using Student’s *t*-test (n = 4). Data are expressed as mean ± s.e.m. *P<0.05; **P<0.01; ***P<0.001. ns = non-significant.

We first confirmed that *Igf-1r* heterozygosis leads to decreased levels of the receptor (Figure 5A). However, this was not associated with any obvious change in the phosphorylation of AKT at T308 (which correlates with its activation activity [39], nor any change in the phosphorylation of the mTORC1 substrate p70S6K in mouse brains between any of the genotypes (Figure 5A). We then tested whether *Igf-1r* deficiency affected autophagosome numbers at this early age by measuring LC3-II levels (as a function of actin). We could not detect any significant changes in the levels of LC3-II in the brains of non-HD or HD mice regardless of their *Igf-1r* levels, although this may be due in part to mouse-to-mouse variability (Figure 5B). We found similar results with the male brains at 12 weeks (Figure S3A) and female lysates at a later time point (18 weeks, Figure S3B). Phosphorylation of AKT at serine 473 (S473), which correlates with mTORC2 activity [39] was not significantly affected in *Igf-1r* deficient females at 12 weeks of age despite having low levels of IGF-1R protein. Indeed, AKT phosphorylation at S473 has a tendency to be diminished (as ratio with total AKT) in Non-HD *Igf-1r^+/−^* females at 18 weeks and in *Igf-1r^+/−^* males at 12 weeks of age (Figure S3A, B). Interestingly, at both time points, no differences were found in AKT phosphorylation at S473 in the HD context regardless of their *Igf-1r* levels, suggesting that HD pathology affects AKT activation independent of *Igf-1r* status. Collectively, these observations suggest that the contribution of IGF-1R to the modulation of the downstream signalling pathways *in* *vivo* is influenced by multiple factors, such as age and sex, and can be influenced by the HD background.

## Discussion

Based on the current literature, there are data supporting both protective and deleterious effects of diminishing IIS pathway in HD models. Thus, perhaps not surprisingly, we found that heterozygous deletion of *Igr-1r* lead to complex behavioural effects in HD mice, with opposite effects in male and female HD mice that did not lead to an overall modification of survival. This was accompanied by unchanged levels of AKT activation and mTOR signalling in the HD mice, leading to no effects in autophagy. Thus, modulating IIS pathway via *Igf-1r* deficiency leads to paradoxical effects in the onset and progression of HD in male and female mice.

*Igf-1r* deficient mice have previously shown sexually dimorphic phenotypes, such as lifespan extension, which is only significant in *Igf-1r* ^+/*−*^ females [33], [36]. This lifespan extension is accompanied with resistance to acute oxidative stress after paraquat injections that are present in *Igf-1r* ^+/−^ females but not *Igf-1r* ^+/−^ males. This is interesting, as here the only beneficial effects of *Igf-1r* deficiency are seen in HD females. However, this model is not generally applicable, because no sexual dimorphism was reported upon *Igf-1r^+/−^* mice 1-methyl-4-phenyl-1,2,3,6-tetrahydropyridine (MPTP) treatment [40] or when crossed to AD mouse models [27]. Thus, the opposite effects of *Igf-1r* deficiency in HD males and females may reflect an inherent difference in IIS between males and females in the C57BL/6J background that could be exacerbated by HD pathology. In this regard, IGF-1 levels have been shown to decrease in an age-dependent manner in the R6/2 HD mouse model, associating IGF-I levels with the body weight loss that occurs in HD patients [41]. However, in the N171-82Q HD mouse model studied here, IGF-1 levels were higher in all HD females regardless of *Igf-1r* gene status and were also elevated in HD; *Igf-1r* ^+/−^ males. These differences may be due to the fact that here IGF-1 levels were measured here at an early disease time point on C57BL/6J genetic background, which has been shown to produce low levels of circulating IGF-1 [42]. High levels of GH (growth hormone) and IGF-1 have been reported in HD patients [19], [43], [44], with no differences in IGF-1 levels between genders. Only in HD male patients, the high levels of IGF-1 were correlated with increased cognitive decline [44].

AKT activation is known to have anti-apoptotic effects and being neuroprotective in Huntington’s disease through the direct phosphorylation of huntingtin at serine 421 [17]. In the N171-82Q mouse model, the transgene expresses the N-terminal 171 residues of human huntingtin with 82 polyglutamines, and therefore lacks serine 421. Thus, the possible neuroprotective effects through the direct phosphorylation of mutant huntingtin are not modelled in this study.

In *C. elegans*, a reduction of IIS has been shown to protect from toxic aggregation in different proteinopathies, including HD [25], [45]. Nonetheless, in the R6/2 HD mouse model, IGF-1 administration has been shown to lead to some beneficial effects, including protection against diabetes and hind-limb clasping, but not on motor function [24]. Recently, administration of IGF-1 has been proven to rescue Huntington’s disease phenotypes in YAC128 mice [23]. These paradoxical beneficial effects of both a reduction and an increase in IIS have also been shown in AD models. In worms and mice, IIS reduction leads to protection from toxicity associated with the aggregation of human Abeta [27], [28], [46]. Interestingly, in one of the studies, diminished expression of *Irs2* in the AD mice protected females but not males [28] just as we observed here. IIS reduction has also been linked to a compaction of the deposited Abeta plaques [27], [46], that could correlate with the changes in huntingtin cerebellar inclusion sizes in the present study. However, as in the HD case, an increase in IIS (by administration of IGF-I) has also been shown to protect from Abeta mediated toxicity [47]. Thus, as is the case for AD, both an increase and a decrease in IIS may have some potential therapeutic value in HD models, perhaps affecting different features of the disease, which could also be influenced by sex, at least in the mouse.

We have recently published that long-term *Igf-1r* depletion or chemical inhibition impaired autophagy, which was unexpected given the literature on short-term effects of IGF1 administration. Our previous *in* *vitro* and *in* *vivo* data suggested that IGF-1R inhibition decreases autophagosome formation by reducing endocytosis, a step which is tightly controlled by the mTORC2 complex activity [32]. However, here we show that the *Igf-1r* hemizygosity (which causes, as expected, a 50% reduction in the receptor levels) does not have an obvious effect on downstream mTOR signalling or autophagosome numbers *in* *vivo*. We have recently proposed that a complex auto-regulatory feedback loop mechanism allows the mTORC1 complex activity to be sustained when IGF-1R activity is reduced in cell-based systems [32]. It is worth noting that, in this context, even with a 80% reduction of the IGF-1R receptor levels, cells were capable of activating the mTORC1 downstream signalling modules upon IGF-1 stimulation [32]. Such a scenario may explain how the mTORC1 pathway activity is quite tightly buffered and controlled in the long term in mammalian systems. Moreover, the mTORC2 activity (as assessed by AKT phosphorylation on serine 473) was overall not affected by *Igf-1r* heterozygosis in the HD mice. It is quite possible that a reduction of IGF-1R signalling of greater than 50% (along the lines we reported previously [32]) may be required to consistently impact on mTORC2 signalling (and autophagy). One should also note that the LC3-II only assess steady-state levels of the protein (which correlates for autophagosomes number), making difficult to infer about any possible and concomitant change in the rate of synthesis and/or flux through the pathway [48]. Hence, we cannot exclude the possibility that the decreased aggregate numbers in the cerebellum of HD; *Igf-1r* hemizygous mice compared to control littermates, may be resulting from differential acute nutrient/IGF1/insulin signalling in the mice that may be sufficient to create a short-term relative difference in autophagy sufficient to impact on huntingtin aggregate numbers, even if the “steady-state” activity of the mTOR pathway were unchanged in these mice.

Overall, altering the very refined pathway of IIS in different proteinopathies leads to differential outcomes in different disease model systems. Intriguingly, both diminishing and increasing IIS pathway seem to lead to some beneficial effects in models of HD, although in HD males, we show here that diminishing IIS could also lead to some deleterious effects.

## Methods

### Ethics statement

All procedures were carried out with the appropriate UK Home Office and MRC Harwell Ethical Committee approval.

### Mice

Transgenic mice expressing the first 171 residues of huntingtin with 82 CAG repeats [49] were maintained in hemizygozity by backcrossing to C57BL/6J (B6) for more than 10 generations (both from Jackson Laboratories). *Igf-1r^+/−^* mice, described elsewhere and available from http://www.emma.rm.cnr.it were maintained as hemizygotes by backcrossing to C57BL/6J. Genotyping was carried out by PCR of DNA from the ear clips, using the primers recommended by the distributor (http://www.emma.rm.cnr.it) To generate double mutants and their appropriate control littermates, we crossed hemizygous N171-82Q (B6 congenic) males to *Igf-1r* deficient females. We always used mice from the first generation for all the analysis performed. Observers were blind to mouse genetic status during testing.

### Behavioural phenotype

We assessed modified SHIRPA, rotarod and grip strength, as previously described [35] analysis every two weeks, from 8 weeks of age to death. A minimum of seven mice per group and gender were analysed for a specific test. Grip strength (BIOSEB, France) and rotarod tests (Accelerating model, Ugo Basile, Italy) were done in alternating weeks with the modified SHIRPA testing. Tremors onset and severity, wire manoeuvre and other tasks are monitored as part of the modified SHIRPA battery of behavioural tests. We also weighed the mice every week and checked survival, defined as time until they reach their humane end-points (in accordance with the Ethical Committee). Humane end-points were defined as the loss of more than 20% of maximum body weight or hunched appearance.

### Immunoblot

Brains were frozen immediately and stored at –80°C from F1 offspring representing all four possible genotypes (doubled mutants and control littermates). We used at least n = 3 animals for each genotype and gender at 12 and 18 weeks of age, to compare an early and late stage of the disease. We added 2.5 volumes of buffer B (50 mM Tris (pH 7.5), 10% glycerol, 5 mM magnesium acetate, 0.2 mM EDTA, 0.5 mM dithiothreitol) or RIPA buffer, with protease inhibitors (complete miniEDTA-free, Roche) and phosphatase inhibitors cocktail (PhosphoStop, Roche), following homogenization in lysing matrix tubes D (MP Biomedicals, Germany) and a Fast-Prep-24 homogenizer at 4°C. Homogenates were then centrifuge at 10000×*g* for 15 min and the supernatant was retained. Protein concentration was determined (Bradford reagent, Sigma) and an equal amount of protein from each sample was resolved on SDS-PAGE (typically 4–12% Bis-Tris NUPAGE precast gels from Invitrogen). After transfer to PVDF membranes (GE Healthcare), membranes were blocked in 5% BSA in TBST (Tris-Buffer solution with 0.1% Tween-20) and incubated with primary antibodies overnight at 4°C. The primary antibodies assessed included rabbit anti-phospho-IGF-IR (Abcam, UK), rabbit polyclonal anti-total IGF-IRβ (Santa Cruz, USA), rabbit phospho-Akt ser427, rabbit Pan AKT, from Cell Signalling; rabbit anti-actin (Sigma), mouse anti-soluble human polyQ (MAB1537-1C2, Millipore), LC3II (Novus). Followed by secondary antibody incubation (Goat anti-rabbit IgG and anti-mouse IgG peroxidase cojungated) for 1–3 hours at room temperature, blots were developed with ECL plus detection kit (GE Healthcare). We carried out densitometry of the films using Image-J software.

### Immunohistochemistry

We carried out immunohistochemistry on brain slices from mice perfused transcardially with 4% (w/v) paraformaldehyde (Sigma) in phosphate saline buffer (PBS) pH 7.4. Coronal brain cryo-sections, ±1 mm from the bregma or the whole cerebellum, at 30 µm thickness were generated to perform free-floating slices staining for inclusions at 13 weeks of age females (n = 4 per HD group). To detect mutant human huntingtin aggregates the primary antibody (MAB5374 from Millipore or the previous EM48 Chemicon) was incubated overnight, followed by secondary mouse Alexa-Fluor488 conjugated antibody (Invitrogen) and mounted on Vectashield with nuclear counterstaining DAPI (Vector labs). Confocal images were taking using Zeiss LSM 700 microscope at 60X amplification of the piriform cortex. Final image stack projections (typically the sum of 12 images per projection) were used to count the total number of inclusions per field. A total of three consecutive fields in the piriform cortex per slice, over 3 or 4 slices per mouse and 4 mice per genotype are used to account for the total number of inclusions in the piriform cortex. The counting was done by counterstaining the nuclei with DAPI, therefore excluding in the majority of the cases the somatodendritic aggregates. After manually counting the inclusions, we used the Volocity software 5.4.1 (Perkin Elmer, USA) on the Z-stack projections to verify the counting obtained and corrected by nuclei’s DAPI+ per image.

To measure the size of the inclusions we used image analysis software (Cell∧D, from Axioscop) over the confocal projection images taking from the granular layer of the cerebellum. We used the cerebellum because in the N171-82Q mouse model, at the selected age of 13 weeks of age, the number of inclusions/aggregates are a lot higher and clearer to detect in the cerebellum when compared to the striatum. Again coronal cryosections at 30 µm were cut and free floating staining against mutant huntingtin protein. A total of 3 confocal images (sum of 12 Z-stack at 60x magnification) adjacent to the peak of the folding granular layer per slice, over 4–5 slices per mouse and 6 mice per genotype were used to account for the analysis of inclusions in the cerebellum. The software classifies the inclusions by size and count the total of a given size range. The great majority of huntingtin inclusions present in the cerebellum of the N171-82Q model are intranuclear, as evidenced by DAPI counterstaining. The data is presented as the percentage of the total aggregates for each particular aggregate size. We classified the inclusion sizes into 4 categories, from the smallest aggregates (0–0.3 µm^2^) to the biggest inclusions found (1.8–4.5 µm^2^).

### Seprion ligand ELISA for polyQ aggregates

We performed Seprion ELISA as previously described [38] to quantify huntingtin aggregation. Briefly, male and female HD mice were killed at 12 weeks of age. After removing the brain, the cerebellum was separated and frozen at –80 until used. Cerebellum homogenates in RIPA buffer with protease inhibitors were subjected to ELISA detection for mutant huntingtin aggregates using MW8 primary antibody and a peroxidase (HRP)-conjugated rabbit anti-goat secondary antibody (DAKO). The product of the reaction after adding the substrate TMB (SerTec) was quantified in a plate reader at 450 nm (Biorad).

### Blood measurements

Blood samples were collected in a heparin-tube by orbital bleed from mice fasted for 4 hours. We used 5 mice at 12 weeks of age per genotype and gender. After spinning, the plasma was used to quantify Igf-1 by ELISA (Mediagnost, Germany) following the manufacturer recommendations. We also used the plasma to quantify glucose.

### Statistics

We determined significance levels for comparisons between groups with Student’s t-test, ANOVA, ANOVA with post-hoc Bonferroni correction, non-parametric Fisher’s test and Log-rank test, where appropriate.

## Supporting Information

Figure S1 Grip-strength tests were performed on at least 5 mice per genotype per sex and time-point: (**A**) females and (**B**) males. No differences were observed between HD; *Igf-1r^+/+^* and HD; *Igf-1r^+/−^* mice at any time point analysed. Error bars represent 1×s.e.m (standard error of the mean).
(TIF)

Figure S2 4-hour fasted glucose measured (mmol/L) in females (**A**) and males (**B**), shows a tendency towards higher glucose levels in HD; *Igf-1r^+/−^* males compared to HD; *Igf-1r^+/^* ^+^ mice, which is not significant (p = 0.24, more than 4 mice per group).
(TIF)

Figure S3 Brain homogenates of the indicated genotypes and time points were subjected to western blot analysis to assess AKT activation at ser473 and autophagosome numbers by means of LC3-II measurement controlled by actin levels. **A.** Western blots of half-brain homogenates from 12 weeks old male mice (n = 3 per group) show that AKT phosphorylation at Ser473, corrected by total levels of AKT, have a trend towards a statistically significant reduction in Non-HD; *Igf-1r^+/−^* when compared to *Igf-1r^+/^* ^+^ controls (p = 0.066), whereas no trend appears when comparing the HD groups: HD; *Igf-1r^+/^* ^+^ and HD; *Igf-1r^+/−^* mice (p = 0.40). No significant differences were found in LC3-II levels corrected by actin for any genotype. **B.** Western blots of brain homogenates from 18 weeks old female mice. AKT phosphorylation at Ser473 again shows a trend towards a reduced ratio in Non-HD; *Igf-1r^+/−^* when compared to Non-HD; *Igf-1r^+/^* ^+^ controls (p = 0.1). Again, non-significant differences appear when comparing the HD groups: HD; *Igf-1r^+/^* ^+^ and HD; *Igf-1r^+/−^* mice (p = 0.7). Not significant differences in LC3-II/actin between any of the genotypes were observed. The gels are representative of the analysis of four different brain samples for each genotype (n = 4 per group). Data are expressed as mean ± 2×s.e.m. ns = non-significant.
(TIF)

Table S1 Summary of phenotypic comparison between male, female and both combined for HD; Igf-1r+/+ and HD; Igf-1r+/*−* mice.
(DOCX)
