# Supplementary figures and images for: Reducing Igf-1r Levels Leads To Paradoxical and Sexually Dimorphic Effects in HD Mice
Source: PLoS One. 2014 Aug 20;9(8):e105595. doi: 10.1371/journal.pone.0105595 (PMC4139380; doi:10.1371/journal.pone.0105595)

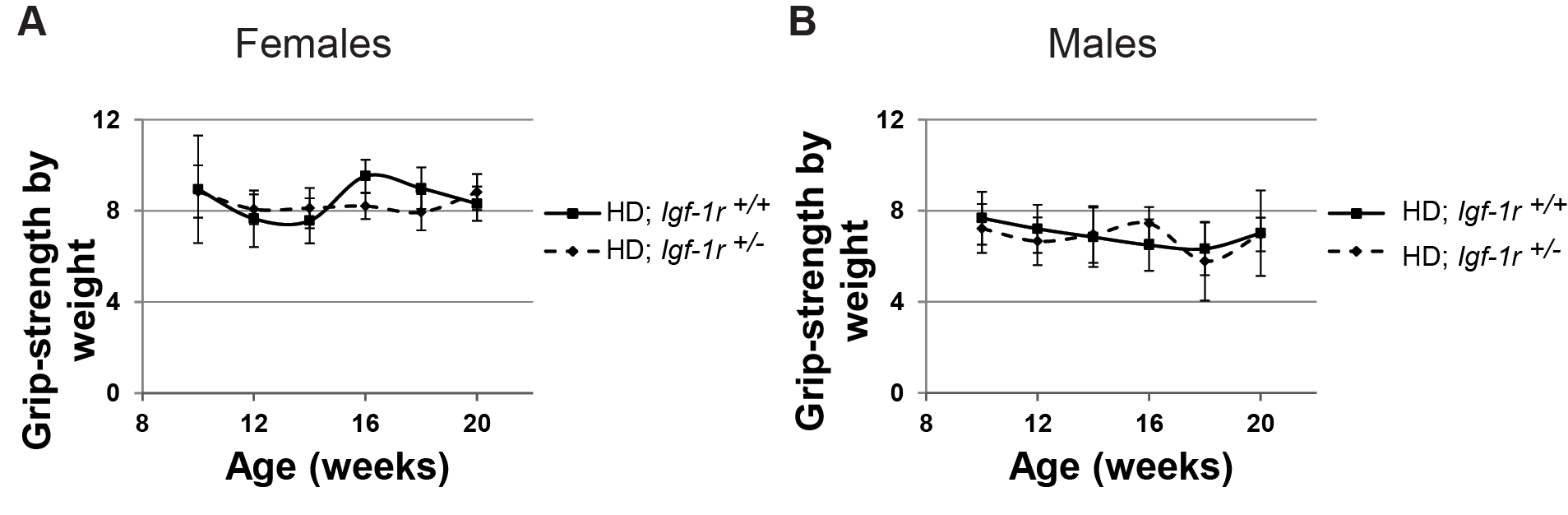

Supplement: Figure S1 — Grip-strength tests were performed on at least 5 mice per genotype per sex and time-point: (A) females and (B) males. No differences were observed between HD; Igf-1r+/+ and HD; Igf-1r+/− mice at any time point analysed. Error bars represent 1×s.e.m (standard error of the mean). (TIF) [file pone.0105595.s001.tif]

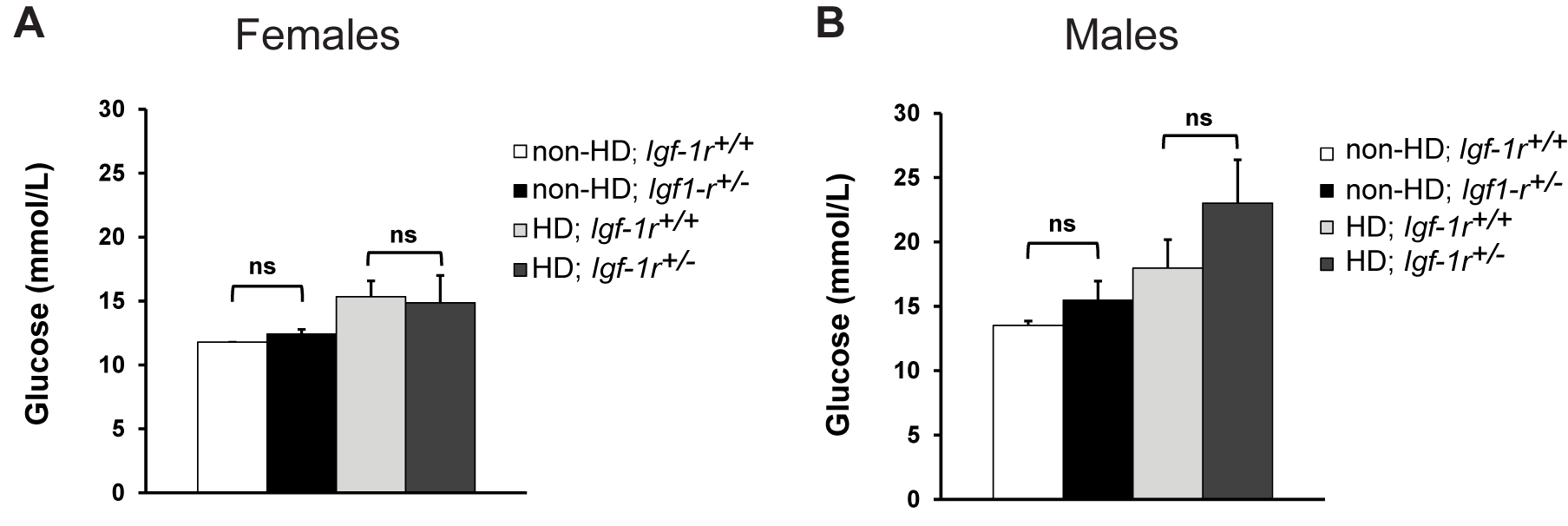

Supplement: Figure S2 — 4-hour fasted glucose measured (mmol/L) in females (A) and males (B), shows a tendency towards higher glucose levels in HD; Igf-1r+/− males compared to HD; Igf-1r+/ + mice, which is not significant (p = 0.24, more than 4 mice per group). (TIF) [file pone.0105595.s002.tif]

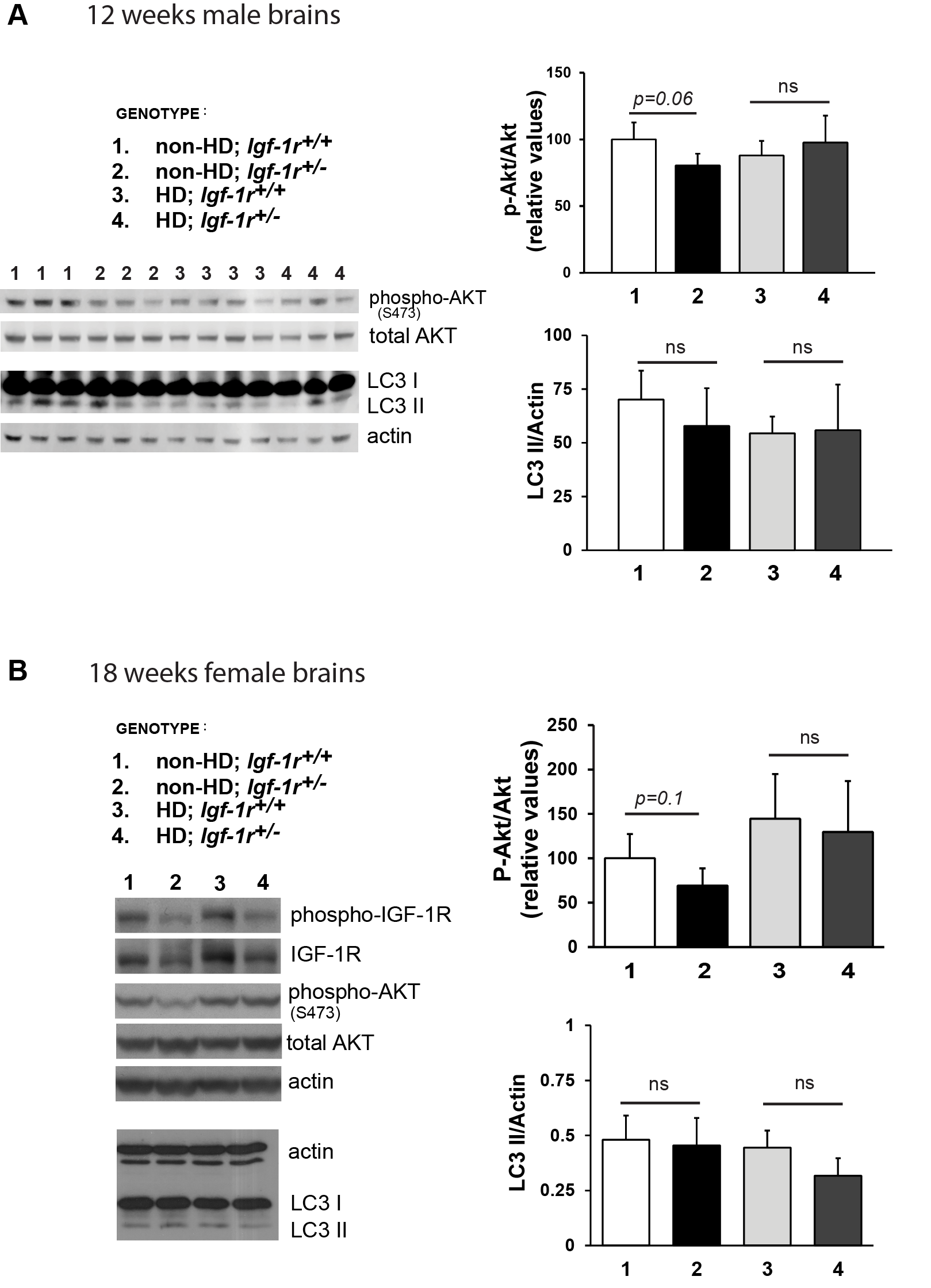

Supplement: Figure S3 — Brain homogenates of the indicated genotypes and time points were subjected to western blot analysis to assess AKT activation at ser473 and autophagosome numbers by means of LC3-II measurement controlled by actin levels. A. Western blots of half-brain homogenates from 12 weeks old male mice (n = 3 per group) show that AKT phosphorylation at Ser473, corrected by total levels of AKT, have a trend towards a statistically significant reduction in Non-HD; Igf-1r+/− when compared to Igf-1r+/ + controls (p = 0.066), whereas no trend appears when comparing the HD groups: HD; Igf-1r+/ + and HD; Igf-1r+/− mice (p = 0.40). No significant differences were found in LC3-II levels corrected by actin for any genotype. B. Western blots of brain homogenates from 18 weeks old female mice. AKT phosphorylation at Ser473 again shows a trend towards a reduced ratio in Non-HD; Igf-1r+/− when compared to Non-HD; Igf-1r+/ + controls (p = 0.1). Again, non-significant differences appear when comparing the HD groups: HD; Igf-1r+/ + and HD; Igf-1r+/− mice (p = 0.7). Not significant differences in LC3-II/actin between any of the genotypes were observed. The gels are representative of the analysis of four different brain samples for each genotype (n = 4 per group). Data are expressed as mean ± 2×s.e.m. ns = non-significant. (TIF) [file pone.0105595.s003.tif]
