# Supplementary material for: Reducing Igf-1r Levels Leads To Paradoxical and Sexually Dimorphic Effects in HD Mice
Source: PLoS One. 2014 Aug 20;9(8):e105595. doi: 10.1371/journal.pone.0105595 (PMC4139380; doi:10.1371/journal.pone.0105595)
Supplement: Table S1 — Summary of phenotypic comparison between male, female and both combined for HD; Igf-1r+/+ and HD; Igf-1r+/− mice. (DOCX) [file pone.0105595.s004.docx]

| Behavioral test | Females | Males | Both combined |
| --- | --- | --- | --- |
|  | p values-Log Rank |  |  |
| Survival | 0.26 (N=10; N=19) ^*^ | 0.48 (N= 15; N=12) | 0.794 (N=25; N=31) |
| Tremor onset | 0.002 (N=8; N=16) | 0.278 (N=11; N=12) | 0.01 (N=19; N=28) |
|  |  |  |  |
|  | p values-ANOVA (Bonferroni post-hoc)** |  |  |
| Rotarod | NS | >0.001 | >0.001 |
| Grip strength | NS | NS | NS |
| Weight | NS | >0.01 | >0.05 |
|  |  |  |  |

**Supplementary Table 1**: Summary of phenotypic comparison between male, female and both combined for HD; Igf-1r+/+ and HD; Igf-1r+/- mice.

^*^  N= number of mice per genotype, first HD; *Igf-1r^+/+^* and second HD; *Igf-1r^+/-^*.

^**^ *p* values referred to time points from 12 weeks of age until 20 weeks of age. NS= Not significant.
